# Landscape complexity influences route-memory formation in navigating pigeons

**DOI:** 10.1098/rsbl.2013.0885

**Published:** 2014-01

**Authors:** Richard P. Mann, Chris Armstrong, Jessica Meade, Robin Freeman, Dora Biro, Tim Guilford

**Affiliations:** 1 Mathematics Department, Uppsala University, Uppsala, Sweden; 2 Department of Engineering Science, University of Oxford, Oxford, UK; 3 Department of Zoology, University of Oxford, Oxford, UK; 4 School of Biological, Earth and Environmental Sciences, University of New South Wales, Sydney, Australia; 5 Institute of Zoology, Zoological Society of London, Regents Park, London, UK

**Keywords:** pigeon, navigation, homing, familiar area, landmark, vision

## Abstract

Observations of the flight paths of pigeons navigating from familiar locations have shown that these birds are able to learn and subsequently follow habitual routes home. It has been suggested that navigation along these routes is based on the recognition of memorized visual landmarks. Previous research has identified the effect of landmarks on flight path structure, and thus the locations of potentially salient sites. Pigeons have also been observed to be particularly attracted to strong linear features in the landscape, such as roads and rivers. However, a more general understanding of the specific characteristics of the landscape that facilitate route learning has remained out of reach. In this study, we identify landscape complexity as a key predictor of the fidelity to the habitual route, and thus conclude that pigeons form route memories most strongly in regions where the landscape complexity is neither too great nor too low. Our results imply that pigeons process their visual environment on a characteristic spatial scale while navigating and can explain the different degrees of success in reproducing route learning in different geographical locations.

## 1. Introduction

Pigeons have long served as a model for the study of avian navigation, and it is known that they can make use of some form of navigational map, probably based on atmospheric odours, combined with time-compensated solar compass information, in a two-step process, to orient homeward from distant unfamiliar places [1]. Once pigeons become familiar with their environment, however, they are thought to resort to the second mechanism of homing, involving memorized local features [2]. The nature of this familiar area map has received far less attention until recently, with the dominant finding being that pigeons released repeatedly from the same site have been observed to form idiosyncratic memorized paths to the home loft [3–5], which they are attracted back to and faithfully recapitulate even if released a short distance from the original site [4], strongly suggesting the use of familiar visual cues. Route formation and route fidelity vary greatly, however, within individual routes, between individuals, between sites and even between lofts [6,7], but the cause of this variation is not understood. Here, we attempt to improve our understanding of the phenomenon by investigating the relationship between the route-memory formation and visual information complexity of the underlying landscape.

Previous analyses of the relationship between these memorized flight paths and the landscape have indicated a preference for prominent linear features in approximately the homeward direction [3,4,8,9]. A naive hypothesis might argue that the bird's ability to determine its position and required direction should increase monotonically with the amount of visual information available, leading to faster learning of the memorized route and greater fidelity in recapitulation. Supporting this idea, previous studies have found that birds are attracted to edge-containing locations in the landscape and change their flight behaviour significantly when approaching them [10]. However, experiments by Wiltschko *et al*. [11] and Schiffner *et al*. [7] have indicated that pigeons may be unable to form a memorized route in a highly urban environment (suburban Frankfurt, Germany). Visual inspection of the positions of probable landmarks in our previous research [12] has also suggested that pigeons may form fewer memories in highly urban areas. Such environments provide a wealth of visual information, but theoretical neural-network models have suggested that excessive information may lead to confusion and difficulty in route learning [13]. This poses the question of whether there is an optimal density of information in the landscape over which a bird is homing for the formation or processing of visual memories. We approach this problem by analysing how the route fidelity varies in relationship to underlying landscape complexity over different stages of the route-learning process.

## 2. Material and methods

Further methodological details are given in the electronic supplementary material.

### (a) Experimental procedures

**Figure 1.**
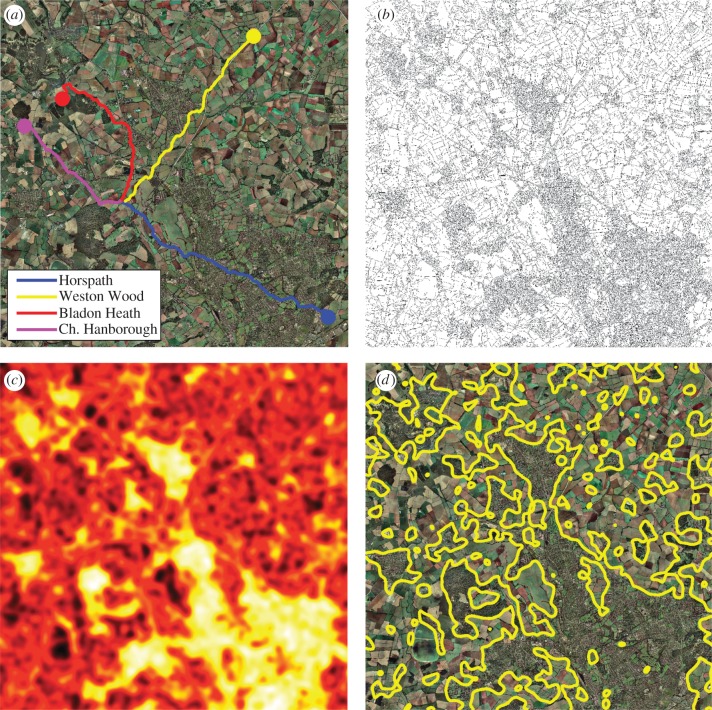
Details of the visual landscape. We analysed the visual information provided by the landscape using an aerial image of the Oxford area (*a*), with a single flight path from each of the four release sites shown to indicate coverage. We filtered this image to detect ‘edges’, defined as sharp changes in image intensity (*b*). The local density of these edges was calculated by evaluating the number of edges in the local area to produce an edge-density map (*c*), which indicates the amount of information in a local region. After determining the optimal edge density by our regression analysis (figure 2), we overlaid a contour of this edge density on the original aerial image (*d*), showing that the optimal density is found on the boundary between urban or forested regions and open rural regions.

Using micro-GPS devices attached to the birds’ backs, we recorded the flight paths of pigeons repeatedly released from one of four release sites in the Oxford area, indicated in figure 1*a*. Every bird was initially naive and was released a total of 20 times from its selected release site.

**Figure 2.**
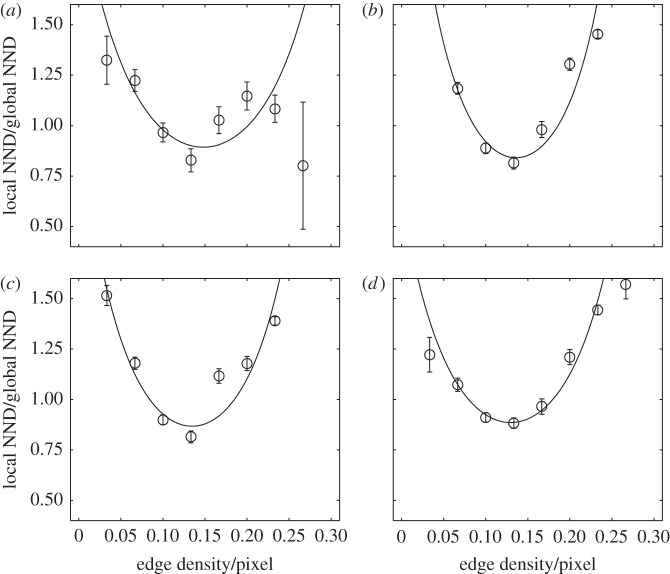
Variation in nearest-neighbour distance (NND) with edge density. Panels indicate different stages of the route-learning and recapitulation process: (*a*) releases 1–5, (*b*) 6–10, (*c*) 11–15 and (*d*) 16–20. Results are shown for edge density calculated over a visual radius of 250 m, which maximizes the quality-of-fit. Edge density is calculated per pixel of the aerial landscape image and each pixel covers a 5×5 m area. Because the global route fidelity varies between different stages of the route-learning process, we show the local NND between paths in proportion to the global average for that experience stage. At all stages, and for all visual areas, a polynomial regression selects a U-shaped quadratic fit (based on the AIC [17]).

This research was conducted in accordance with UK animal welfare legislation and the most recent edition of the ASABs Guidelines for the treatment of animals in behavioural research and teaching.

### (b) Estimating visual information

We defined the amount of visual information in the landscape by the local density of edges in an aerial image. We used the canonical Canny edge-detection filter [14] to identify edges. We then took localized averages of the number of pixels containing edges at each point in the image. We performed this averaging over a range of scales from 100 to 500 m to ensure that our results were not sensitive to a particular visual range of the birds.

### (c) Calculating route fidelity

We defined the route fidelity of a bird by the average proximity of one flight path to that immediately preceding it. For every pair of successive paths, we calculated the nearest-neighbour distance (NND) from every point on the first path to its closest neighbouring point on the second path. Smaller distances between successive paths indicate greater fidelity to a memorized path.

### (d) Data availability

The GPS tracking data and aerial landscape image used in this study are provided as a supplementary archive.

## 3. Results

Our previous research has consistently confirmed the route-following phenomenon [3–5,12]. However, many of the release sites used around the Oxford Field Station are in relatively rural locations. In the light of the results of earlier studies [7,11], we decided to investigate whether highly urban environments systematically inhibited route learning, by re-analysing in detail the relationship between how well birds recapitulate their habitual routes and the visual properties of the landscape. We used data from previous studies [5,12] consisting of initially naive birds released repeatedly from four different sites between 5.0 and 10.6 km from the Oxford loft (shown in figure 1*a*). We defined measures of local visual information density in the landscape and route fidelity in the flight paths of the birds (see Material and methods) and assessed how the route fidelity was related to both the number of times the bird had previously been released from the site and the amount of local visual information.

We measured the route fidelity in this study by calculating the distance between each point on one flight path and its nearest-neighbouring point on the next, providing a measure of route fidelity at each location at which the bird was recorded. Previous studies on these data showed that the average measured route fidelity of the birds in this study increases with release number [5,12] (and see the electronic supplementary material, figure S1), demonstrating the increasing dependence of the flight paths upon habitual route memories. Illustrative examples of the analysed flight paths are shown in both [5] and [12].

To determine quantitatively how these route memories relate to the information content of the underlying landscape, we analysed an aerial image of the Oxford area encompassing all the release sites, flight paths and the home loft. We processed this to find the local information density at each point in the image, in terms of the density of edges, which have been shown to constitute the fundamental aspect of animal vision and the most statistically independent components of natural images [15,16]. We calculated the density of these edges over a variety of area sizes, to account for uncertainty over the perception characteristics of the birds.

Incorporating all the data from the four release sites (31 birds, 620 flight paths), we calculated the NND from each path to its successor, obtaining a measure of fidelity for each point in space that the bird visits during its first 19 flights. We split these data into four experience stages, corresponding to the first 5, second 5, third 5 and final 5 flights. For each of these phases, we plotted the variation of NND between paths with the underlying edge density in the landscape (figure 2). The NNDs are averaged over 10 bins, corresponding to different edge densities. Visual inspection of these plots suggests that the route fidelity is the greatest at intermediate edge densities. To confirm this, we performed a polynomial regression of the NND on the edge density. In all four experience stages, and for all possible visual areas, the regression analysis selected a quadratic function based on Aikeke Information Criterion (AIC values) [17]. This indicates that the route fidelity does not either increase or decrease monotonically with the edge density, but instead the distance between paths follows a ‘U-shaped’ fit, confirming the existence of an optimal edge density. This fit improved with each experience stage, as can be seen visually in figure 2, especially after the first stage. Our regression analysis indicates that the route fidelity is best predicted by the underlying edge density over regions of approximately 250 m in radius (see electronic supplementary material), which suggests a similar-sized area over which the birds perceive patterns in the landscape.

## 4. Discussion

Our results indicate a gradual increase in the birds’ ability to return to familiar locations with an increasing edge density in the low-density regime, suggesting that visual information is important in the ability to form localized route memories. An optimum point is reached at approximately 0.125 edges per pixel, implying a 12.5% chance for each 5 × 5 m region in the local area covered by an aerial image pixel to contain an edge. Beyond this point, there is a decline in route fidelity with increasing edge density. This supports the idea that pigeons have a reduced ability (or reduced preference) to memorize or process visual information that is too dense. By maximizing the fit between the calculated edge density and resulting route fidelity using different visual areas, we were able to identify the scale on which the birds process the visual landscape information. The improving regression fit between the edge density and route fidelity with each learning phase may indicate that pigeons form initial, low-precision memories in highly dense regions but do not then improve on these memories as their routes become more developed.

We overlaid the edge-density map, calculated using this optimal averaging area, on the landscape image, and established a contour marking the areas within which edge density was greater than this optimum. As figure 1*d* shows, this contour almost perfectly outlines the urban areas in the landscape image, as well as including a number of areas of forest, which pigeons have also been observed to avoid flying over [18]. This further supports the idea that pigeons may be less able to memorize route information in urban environments. It is not obvious whether this is because urban areas are just beyond this optimal point in terms of information density or whether the optimum is fixed at this value because it coincides with the boundary of urban environments. The boundary between urban and rural areas may provide extremely strong visual information on very large scales that is available to the bird from a distance away, which may explain its attractiveness for forming route memories.

The identification of an optimal information density in the visual landscape also suggests that pigeons’ familiar routes home from specific release sites may be at least in part predictable *a priori*. According to our findings, initially highly variable homeward routes will tend to become less variable where they happen to be in regions of optimal edge density. Combining this knowledge with an appropriate model for initial route generation could identify the most probable routes that the birds will eventually memorize.

## Funding statement

R.P.M. was funded by an EPSRC doctoral training grant and an ERC grant to David Sumpter (IDCAB 220/104702003); C.A. was supported by a BBSRC doctoral training grant and D.B. by a Royal Society University Research Fellowship.
